# Ethnic differences translate to inadequacy of high-risk screening for gestational diabetes mellitus in an Asian population: a cohort study

**DOI:** 10.1186/1471-2393-14-345

**Published:** 2014-10-02

**Authors:** Yap-Seng Chong, Shirong Cai, Harvard Lin, Shu E Soh, Yung-Seng Lee, Melvin Khee-Shing Leow, Yiong-Huak Chan, Li Chen, Joanna D Holbrook, Kok-Hian Tan, Victor Samuel Rajadurai, George Seow-Heong Yeo, Michael S Kramer, Seang-Mei Saw, Peter D Gluckman, Keith M Godfrey, Kenneth Kwek, on behalf of the GUSTO study group

**Affiliations:** Department of Obstetrics & Gynaecology, Yong Loo Lin School of Medicine, National University of Singapore, National University Health System, 1E Kent Ridge Road, NUHS Tower Block, Singapore, 119228 Singapore; Saw Swee Hock School of Public Health, National University of Singapore, 16 Medical Drive, Singapore, 117597 Singapore; Department of Paediatrics, Yong Loo Lin School of Medicine, National University of Singapore, National University Health System, 1E Kent Ridge Road, NUHS Tower Block, Singapore, 119228 Singapore; Singapore Institute for Clinical Sciences, Agency for Science and Technology Research (A*STAR), Brenner Center for Molecular Medicine, 30 Medical Drive, Singapore, 117609 Singapore; Department of Endocrinology, Tan Tock Seng Hospital, 11 Jalan Tan Tock Seng, Singapore, 308433 Singapore; Office of Clinical Sciences, Duke-NUS Graduate Medical School, 8 College Road, Singapore, 169857 Singapore; Biostatistics Unit, Yong Loo Lin School of Medicine, National University of Singapore, National University Health System, 1E Kent Ridge Road, NUHS Tower Block, Singapore, 119228 Singapore; Department of Obstetrics and Gynaecology, KK Women’s and Children’s Hospital, 100 Bukit Timah Road, Singapore, 229899 Singapore; Department of Neonatology, KK Women’s and Children’s Hospital, 100 Bukit Timah Road, Singapore, 229899 Singapore; Department of Maternal Fetal Medicine, KK Women’s and Children’s Hospital, 100 Bukit Timah Road, Singapore, 229899 Singapore; Department of Epidemiology, Biostatistics and Occupational Health and Department of Pediatrics, McGill University Faculty of Medicine, 845 Rue Sherbrooke Ouest, Montréal, QC H3A 0G4 Canada; Liggins Institute, University of Auckland, Auckland, 1142 New Zealand; Medical Research Council Lifecourse Epidemiology Unit, Southamptom, SO16 6YD UK; NIHR Southampton Biomedical Research Centre, University of Southampton and University Hospital Southampton NHS Foundation Trust, Southampton, SO16 6YD UK

**Keywords:** Universal screening, High risk screening, Gestational diabetes, Asians, Ethnic

## Abstract

**Background:**

Universal and high-risk screening for gestational diabetes mellitus (GDM) has been widely studied and debated. Few studies have assessed GDM screening in Asian populations and even fewer have compared Asian ethnic groups in a single multi-ethnic population.

**Methods:**

1136 pregnant women (56.7% Chinese, 25.5% Malay and 17.8% Indian) from the Growing Up in Singapore Towards healthy Outcomes (GUSTO) birth cohort study were screened for GDM by 75-g oral glucose tolerance test (OGTT) at 26–28 weeks of gestation. GDM was defined using the World Health Organization (WHO) criteria. High-risk screening is based on the guidelines of the UK National Institute for Health and Clinical Excellence.

**Results:**

Universal screening detected significantly more cases than high-risk screening [crude OR 2.2 (95% CI 1.7-2.8)], particularly for Chinese women [crude OR = 3.5 (95% CI 2.5-5.0)]. Pre-pregnancy BMI > 30 kg/m^2^ (adjusted OR = 3.4, 95% CI 1.5-7.9) and previous GDM history (adjusted OR = 6.6, 95% CI 1.2-37.3) were associated with increased risk of GDM in Malay women while GDM history was the only significant risk factor for GDM in Chinese women (adjusted OR = 4.7, 95% CI 2.0-11.0).

**Conclusion:**

Risk factors used in high-risk screening do not sufficiently predict GDM risk and failed to detect half the GDM cases in Asian women. Asian women, particularly Chinese, should be screened to avoid under-diagnosis of GDM and thereby optimize maternal and fetal outcomes.

**Electronic supplementary material:**

The online version of this article (doi:10.1186/1471-2393-14-345) contains supplementary material, which is available to authorized users.

## Background

Gestational diabetes mellitus (GDM), even when mild, can have adverse consequences on the health of both mother and child during pregnancy and early postpartum period [1, 2]. Women with GDM have increased risk of GDM recurrence in subsequent pregnancies [3–5], and of overt type 2 diabetes mellitus [4, 6], while infants born to mothers with GDM are at increased risk of developing obesity and diabetes in later life [7]. Studies have shown that screening, detection and management of GDM can mitigate the risk of adverse outcomes and metabolic diseases in both mother and child [8–10]. The development of GDM may thus be an indicator of subsequent metabolic risk that requires identification and appropriate management.

Factors such as family history of diabetes mellitus, maternal obesity, age >30 years and GDM in previous pregnancies have been reported to increase the risk of GDM [11]. Although the diagnostic criteria are still debated, the oral glucose tolerance test (OGTT) is generally recognized as the ‘gold standard’ for diagnosis of GDM [12].

It is controversial whether screening for GDM with an OGTT in pregnant women should be universal (testing all pregnancies) or selective (testing only pregnant women with risk factors for GDM) [13, 14]. American Diabetes Association, American College of Obstetricians and Gynecologists, UK National Institute for Health and Clinical Excellence (NICE) recommend screening in women with known risk factors [15–17] while other organizations like International Association of Diabetes and Pregnancy Study Groups (IADPSG) support universal screening at 26–28 weeks gestation [18]. Results from several studies indicate that universal screening for GDM in certain populations is more sensitive and cost-effective [14, 19, 20], especially in terms of preventing future type 2 diabetes.

Asians are at greater risk of GDM compared to their Caucasian counterparts [21, 22]. In Singapore, the three major ethnic groups are all Asian: Chinese, Malay and Indian. These three ethnicities jointly comprise over 40% the global population [23]. To the best of our knowledge, our study is the first to include these three ethnic groups in a single cohort and to compare the proportion of GDM using universal versus selective (high-risk for GDM) screening among them.

## Methods

Pregnant women 18 years and above, who were in their first trimester were recruited from the KK Women’s and Children’s Hospital (KKH) and National University Hospital (NUH) in Singapore between June 2009 and September 2010 to participate in the Growing Up in Singapore Towards healthy Outcomes (GUSTO) birth cohort study. Our study included singletons and multiple pregnancies but only for the pregnancy when they were recruited. These women were either Singapore citizens or permanent residents from three different ethnic groups with homogenous parental ethnic background (both parents of the same ethnic group): Chinese, Malay, and Indian. Women who were on chemotherapy, psychotropic drugs or those with type 1 diabetes were excluded [24]. Demographic data, family and obstetric history of the subjects were obtained from interviewer-administered questionnaires at the time of enrolment. Maternal pre-pregnancy weight was self-reported. The study received approval from Institutional Review Board (IRB) that reviews ongoing research in KKH and NUH (Centralized Institutional Review Board and Domain Specific Review Board respectively). Informed written consent was obtained from each participant.

Testing for GDM was performed using a 75-g oral glucose tolerance test after overnight fasting (8 to 10 hours) at 26–28 weeks gestation. The 1999 World Health Organization (WHO) standard criteria were selected for the study define GDM: ≥7.0 mmol/L for fasting glucose and/or ≥7.8 mmol/L for 2-hour post-glucose [25, 26] as blood glucose levels were only collected twice (fasting and 2-hour post-glucose) for our study, to minimize subject burden. Women with GDM were subsequently managed according to standard protocols practiced across both hospitals.

Participants were stratified according to risk factors for GDM to compare the performance of a high-risk screening approach to universal screening for detecting GDM. Subjects were categorized as high-risk for GDM based on the guidelines recommended by UK NICE if they fell into one or more of the following categories: (1) obese [body mass index (BMI) of >30.0 kg/m^2^], (2) family history of type 2 diabetes (first-degree relative), (3) GDM in a previous pregnancy, (4) previous delivery of a baby with a birth weight of ≥4.5 kg or (5) ethnic origin with known high prevalence of diabetes (South Asians, black Caribbean, Middle Eastern) [17]. As South Asian includes anyone of family origin from India, Pakistan or Bangladesh, all Indian subjects were classified as high-risk.

The McNemar chi-square test was performed to compare the prevalence of detected GDM based on universal versus high-risk screening using. Ordinary chi-square test was utilized to compare the prevalence of detected GDM across the ethnic groups. Logistic regression was used to calculate the odds of GDM associated with each of the risk factors used in high-risk screening. The odds ratios for comparison across ethnic groups, were further adjusted for pre-pregnancy BMI > 30 kg/m^2^, family history of diabetes, previous history of GDM and macrosomia. Kruskal Wallis and Mann–Whitney U tests were applied to examine differences in quantitative variables, and chi-square test for categorical variables. There are missing data on covariates [pre-pregnancy BMI (8.9%), family history of diabetes (2.7%), previous history of births ≥4.5 kg (3.2%) and GDM (3.1%)]. Multiple imputations of these variables using Chained Equations imputation (20 imputations) yielded very similar findings (data available upon request). As such, missing data on covariates were excluded from the analysis. All analyses were carried out using SPSS version 20.0 (IBM, Armonk, NY, USA).

## Results

From June 2009 to September 2010, 1247 women were enrolled in GUSTO birth cohort. Of these, 1136 (91.1%) received the OGTT at 26–28 weeks gestation; the remaining 111 subjects either declined OGTT or missed their 26–28 weeks clinic visit. No significant differences were found between the 1136 women who had OGTTs and the 111 women who did not have OGTT in terms of ethnicity (with OGTT- 56.7% Chinese, 25.5% Malay and 17.8% Indian; without OGTT- 54.3% Chinese, 24.8% Malay, 20.1% Indian), age (mean ± SD, 30.7 ± 5.1 vs 30.6 ± 5.8 years), parity (46.0% vs 40.2% primiparous), pre-pregnancy BMI (22.7 ± 4.4 vs 22.8 ± 4.6 kg/m^2^), previous history of GDM (3.4% vs 5.6%), family history of diabetes (29.4% vs 23.9%) as well as previous births ≥4.5 kg (0.3% for both). The demographic and clinical characteristics of all 1136 participants who completed the 75-g OGTT are summarized in Table 1. The predominant ethnic group was Chinese (56.7%), followed by Malay (25.5%) and Indian (17.8%). We oversampled Malay and Indian participants in order to have ample statistical power to study the effect of ethnicity. The Chinese women in this study were older and less obese compared to the Malay and Indian women. They were also more likely to be primiparous and less likely to have family history of diabetes.

**Table 1:** **Maternal demographic and clinical characteristics of participants completing the 75-g oral glucose tolerance test** ^**a**^

	Total (n = 1136)			P value*
	Chinese (n = 644)	Malay (n = 290)	Indian (n = 202)	
Maternal age, y	31.7 ± 4.9	29.0 ± 5.5	29.7 ± 4.6	<0.001
Primiparous	308 (47.8%)	116 (40.0%)	73 (36.1%)	0.005
Pre-pregnancy BMI, kg/m^2^	21.6 ± 3.4	24.4 ± 5.6	23.9 ± 4.4	<0.001
BMI > 30 kg/m^2^	17 (2.6%)	41 (14.1%)	18 (8.9%)	<0.001
BMI 25–30 kg/m^2^	68 (10.6%)	64 (22.0%)	49 (24.3%)	
BMI <25 kg/m^2^	501 (77.8%)	158 (54.5%)	119 (58.9%)	
Missing data	58 (9.0%)	27 (9.3%)	16 (7.9%)	
Family history of diabetes mellitus	156 (24.8%)	87 (31.0%)	91 (46.7%)	<0.001
Previous history of GDM	24 (3.8%)	6 (2.1%)	9 (4.6%)	0.288
Previous birth ≥4.5 kg	3 (0.5%)	1 (0.4%)	0 (0%)	0.626
One or more NICE risk factors except ethnicity	177 (27.5%)	117 (40.3%)	101 (50.0%)	<0.001
Fasting plasma glucose ≥7.0 mmol/L	1 (0.2%)	3 (1.0%)	1 (0.5%)	0.170
2 h post OGTT Plasma glucose ≥7.8 mmol/L	135 (21.0%)	34 (11.7%)	45 (22.3%)	0.001

*Abbreviations*: BMI, Body Mass Index; GDM, gestational diabetes mellitus; NICE, UK National Institute for Health and Clinical Excellence; OGTT, oral glucose tolerance test.

^a^Values are given as n (%) or mean ± SD.

*Analysed by Kruskal-Wallis test and chi-square test for qualitative variables categorical variables, respectively.

When the NICE guidelines for high-risk screening were applied to GUSTO, 496 of the 1136 participants who underwent OGTT, were classified as high-risk. Universal screening detected a higher proportion of GDM (18.9%, n = 215) than high-risk screening (9.8%, n = 111) (Table 2). In other words, if OGTT had been performed only in women considered high-risk by the NICE guidelines, almost half of the women with GDM would have been missed.

**Table 2:** **Proportion of detected gestational diabetes mellitus (GDM) in GUSTO cohort using universal versus high-risk screening** ^**a**^

Screening methods	Whole cohort	Chinese	Malay	Indian
	(n = 1136)	(n = 644)	(n = 290)	(n = 202)
Universal^b^	215 (18.9)	135 (21.0)	35 (12.1)	45 (22.3)
High-risk^b^	111 (9.8)^c^	45 (7.0)^c^	21 (7.2)^c^	45 (22.3)

^a^Values are given as number (%).

^b^Universal screening is where all participants undergo oral glucose tolerance test (OGTT); High-risk screening is where only participants with one or more of the risk factors based on the guidelines from UK National Institute for Health and Clinical Excellence (NICE) have to undergo OGTT.

^c^P < 0.001 compared to Universal Screening.

As shown in Table 2, universal screening resulted in GDM prevalence of 21.0%, 12.1% and 22.3% in Chinese, Malay and Indian women respectively. GDM risk based on universal screening remained higher in Chinese (adjusted OR = 2.2, 95% CI 1.4-3.4) and Indian (adjusted OR = 2.2, 95% CI 1.3 -3.6) women relative to Malay women, after adjusting for non-ethnicity related risk factors (Additional file 1: Table S1). Overall sensitivity and specificity of high-risk screening were 51.6% and 52.2% respectively (Additional file 1: Table S2). Sensitivity of high-risk screening is 33.3% in Chinese and 60.0% in Malay; specificity is 74.1% and 62.4% respectively in Chinese and Malay women. Indian women were all classified high-risk by NICE guidelines because of their South Asian origin.

Pre-pregnancy BMI > 30 kg/m^2^ (OR = 3.3, 95% CI 1.5- 7.6) and previous GDM history (OR = 7.6, 95% CI 1.5- 39.2) were associated with increased risk of GDM in Malay women, even after adjusting for all other risk factors (Table 3). In Chinese women, only previous history of GDM was associated with increased GDM risk (adjusted OR = 4.7, 95% CI 2.0-11.0) while none of the non-ethnic risk factors was significantly associated with GDM risk in Indians. Indian ethnic origin was associated with a non-significant increase in GDM risk [adjusted OR = 1.25 (95%CI 0.84-1.86)]. If the pre-pregnancy BMI cut-off was revised to >25 kg/m^2^ to include the overweight women, it is found to be associated with increased risk of GDM in both Chinese (adjusted OR = 2.0, 95%CI 1.2- 3.3) and Malay (adjusted OR = 3.3, 95% CI 1.5-7.1) women (data not shown in table). In addition, maternal age (>25 years) was found to be associated with increased GDM risk only in Malay women (adjusted OR = 7.7, 95% CI 1.01-58.6) (data not shown in table).

**Table 3:** **Ratios for associations between gestational diabetes mellitus and risk factors among the three ethnic groups**

Risk factor	Unadjusted odds ratio (95% CI)			Adjusted odds ratios (95% CI) ^a^		
	Chinese	Malay	Indian	Chinese	Malay	Indian
Pre-pregnancy BMI >30 kg/m^2^	2.7 (1.0-7.2)	3.3 (1.5-7.6)^b^	1.8 (0.6-5.0)	2.4 (0.8-6.8)	3.4 (1.5-7.9)^b^	1.8 (0.6-5.1)
Family history of diabetes	1.0 (0.6-1.5)	1.6 (0.8-3.3)	1.2 (0.6-2.3)	0.8 (0.5-1.3)	1.6 (0.7-3.5)	1.2 (0.6-2.5)
Previous history of GDM	4.8 (2.1-11.0)^b^	7.6 (1.5-39.2)^b^	3.0 (0.8-11.6)	4.7 (2.0-11.0)^b^	6.6 (1.2-37.3)^b^	2.8 (0.7-11.0)
Previous birth ≥4.5 kg	1.9 (0.2-20.9)	Not calculable*	Not calculable*	3.9 (0.2- 62.9)	Not calculable*	Not calculable*

*Abbreviations*: BMI, Body Mass Index; GDM, gestational diabetes mellitus.

^a^Adjusted for other risk factors including pre-pregnancy BMI >30 kg/m^2^, family history of diabetes, previous history of GDM or birth weight ≥4.5 kg.

^b^P < 0.05 *Insufficient number of previous birth ≥ 4.5 kg to calculate.

In this cohort, only 0.4% of the mothers were diagnosed positive for GDM based on the fasting glucose WHO cut-off, compared to 18.5% with the 2-hour post-glucose cut-off. The low diagnosis rate with the fasting glucose was consistent across all the ethnic groups (0.2%, 1.0% and 0.5% in Chinese, Malay and Indian women respectively).

## Discussion

Universal screening at 26–28 weeks gestation in a population of Singaporean women of Chinese, Malay and Indian ethnic origin, detected almost twice as many GDM cases as a high-risk screening approach based on the NICE guidelines. Individual risk factors used in high-risk screening showed poor sensitivity for GDM risk, particularly among Indian women, in whom none of the risk factors was associated with a significant increase in GDM risk. Previous history of GDM significantly increased risk of GDM in both Malay and Chinese women, but it is important to note that many of these women (47.8% and 40.0% in Chinese and Malay women respectively) were primiparous. While other studies have also shown that risk factors are not independent predictors of GDM [27, 28] in Asian populations, they have not compared these predictors among distinct Asian ethnic groups.

Using the WHO criteria, the majority of GDM cases were detected by the 2 h post-glucose levels across all three ethnic groups in this cohort. Fasting glucose levels were relatively low (mean = 4.37 mmol/L, standard deviation = 0.54) whereas 2 h post-glucose levels (mean = 6.57 mmol/L, standard deviation =1.61) were higher than those reported in other ethnicities (South Asian, Middle Eastern, Pacific Islander and Anglo-European) [29]. Monnier *et al.* [30] previously reported that the mean glycemic control was predominantly contributed by postprandial glucose level in patients with mild diabetes compared to standard type 2 diabetes where the fasting plasma glucose contributed more to the glycemic control. This may imply that the GDM observed in our population was a milder form of diabetes which was more effectively picked up with the 2 h post-glucose levels.

Many studies have reported a strong association between maternal obesity and the development of GDM [31–34]. In our cohort, Malay women had a higher rate of obesity than Indian or Chinese women. Yet universal testing indicated that Malay women had a lower prevalence of GDM compared to either of the other two ethnic groups. The reasons for this finding are unclear but we speculate that it may be related to the differential distribution of adipose tissue in different ethnic groups [35] and/or differences in insulin resistance and β-cell function [36, 37]. In view of these substantial physiological differences across the ethnic groups, high-risk screening will not work well in detecting GDM in a multi-ethnic Asian population like Singapore.

The main strength of our study is the inclusion of three Asian ethnic groups in our cohort. These ethnic groups reflect more than 40% of the global population [23]. While many studies have compared universal and high-risk screening for GDM, few have been conducted in Asian populations and none has compared the three ethnic groups included in our study. One limitation of our study is the lack of 1 h post-OGTT plasma glucose reading, hence we used the 1999 WHO diagnostic criteria instead of the revised 2013 WHO diagnostic criteria [38]. We also acknowledge that our cohort may not be representative of the Singapore population, as the participants were recruited from only two hospitals. However, those are the two largest maternity hospitals in the country and included both private and subsidised patients. Selection bias may therefore influence the prevalence of GDM observed among study participants but should not bias associations between GDM and ethnicity or any other risk factors. Another limitation is that pre-pregnancy BMI was self-reported as participants were already pregnant at enrolment. Finally, we were unable to examine the possible consequences of missed GDM detection, because (for ethical reasons) every GDM case was referred for treatment.

Our findings suggest that clinical risk factors used in defining high-risk population are poorly sensitive for predicting GDM risk in the ethnic groups studied, particularly for Chinese women. More than half of the GDM cases would have been missed if high-risk screening was used. Universal testing appears more appropriate for detecting GDM in Asian populations. Although Indian women are known to be at greater risk of GDM, routine screening of Indian women is still not widely practiced in Asia. With increasing migration of Asians to countries like Australia, United States, Canada and United Kingdom, our findings have important implications for screening strategies in those countries as well [39].

Beneficial effects have been reported on both maternal and fetal outcomes with detection and treatment of even mild GDM [40–45]. Crowther *et al*. [40] demonstrated that mothers with GDM randomised into an active intervention group gave birth to infants with lower rate of serious perinatal complications compared to routine-care group. Kwik *et al.* [43] reported observational associations between untreated GDM and macrosomia, shoulder dystocia and preeclampsia. In another observational study among women treated for mild GDM, higher fasting glucose during initiation of diet therapy was associated with increased neonatal fat mass and elevated C-peptide [46]; during the last two weeks before delivery, higher fasting glucose was associated with macrosomia, large for gestational age, and elevated C-peptide [46]. Reduction of adverse outcomes with detection and treatment of even mild GDM can be cost-effective and reduces economic burden on health care [47]. The United States Preventive Services Task Force recently published their recommendation of screening all asymptomatic women for GDM after 24 weeks of gestation, in view of the moderate net benefit of GDM screening in reducing maternal and fetal complications [48].

GDM has adverse long-term consequences for both mother and offspring. Women with GDM have increased risk of developing GDM in a subsequent pregnancy [3–5] and type 2 diabetes within 5–10 years [4]. In addition, children born from pregnancies affected by glucose intolerance have higher risks for obesity and type 2 diabetes in early adult life [3, 4, 7, 49]. Detection of GDM is important to help identify high-risk women and their offspring prior to the clinical onset of adverse consequences; appropriate interventions could reduce their future risk of type 2 diabetes and cardiovascular disease [50].

## Conclusions

Our study suggests that universal screening for GDM should be instituted in the Singapore population, particularly for Chinese and Indian women. In the population we studied, selective screening failed to detect nearly half the women with GDM. Timely detection of even mild GDM is important to prevent adverse outcomes for both mother and offspring. The current practice of high-risk screening for GDM in most Asian countries will miss many cases and thereby hinder prevention of its short- and long-term adverse sequelae.

## Authors’ information

Yap-Seng Chong and Shirong Cai joint first authors.

## Electronic supplementary material

Additional file 1: Table S1: Odds ratio of gestational diabetes mellitus (GDM) detection across ethnic groups using universal and high-risk screening. **Table S2.** Sensitivity and specificity of high-risk screening across ethnic groups. (DOCX 13 KB)
