# Supplementary material for: Ethnic differences translate to inadequacy of high-risk screening for gestational diabetes mellitus in an Asian population: a cohort study
Source: BMC Pregnancy Childbirth. 2014 Oct 2;14:345. doi: 10.1186/1471-2393-14-345 (PMC4190487; doi:10.1186/1471-2393-14-345)
Supplement: Supplementary file 1 — Additional file 1: Table S1: Odds ratio of gestational diabetes mellitus (GDM) detection across ethnic groups using universal and high-risk screening. Table S2. Sensitivity and specificity of high-risk screening across ethnic groups. (DOCX 13 KB) [file 12884_2014_1209_MOESM1_ESM.docx]

**Additional file 1: Table S1**

Odds ratio of gestational diabetes mellitus (GDM) detection across ethnic groups using universal and high-risk screening.

| **Screening Method** | **Unadjusted OR** | | | **Adjusted OR** ^a^ | | |
| --- | --- | --- | --- | --- | --- | --- |
|  | **Chinese** | **Malay** | **Indian** | **Chinese** | **Malay** | **Indian** |
| **Universal** | 1.9 (1.3-2.9) | 1.0 (Ref) | 2.1 (1.3-3.4) | 2.2 (1.4-3.4) | 1.0 (Ref) | 2.2 (1.3-3.6) |
| **High-Risk** | 1.6 (0.9-2.8) | 1.0 (Ref) | 1.3 (0.7-2.3) | 1.6 (0.8-3.1) | 1.0 (Ref) | 1.6 (0.8-3.0) |

Data presented as odds ratio (OR) (95% confidence interval).Ref-Reference group.

^a^ Adjusted for family history of diabetes, pre-pregnancy body mass index, previous history of GDM and previous birth≥4.5kg,

**Additional file 1: Table S2**

**Sensitivity and specificity of high-risk screening across ethnic groups.**

| **High-Risk Screening** |  | **Whole cohort** | | **Chinese** | | **Malay** | | **Indian** | |
| --- | --- | --- | --- | --- | --- | --- | --- | --- | --- |
|  |  | **GDM** | **Non-GDM** | **GDM** | **Non-GDM** | **GDM** | **Non-GDM** | **GDM** | **Non-GDM** |
|  | **Yes** | 111 | 385 | 45 | 132 | 21 | 96 | 45 | 157 |
|  | **No** | 104 | 536 | 90 | 377 | 14 | 159 | Not applicable ^a^ | |

GDM- gestational diabetes mellitus.

Sensitivity and specificity of high-risk screening- 51.6%, 58.2% (whole cohort); 33.3%, 74.1% (Chinese); 60.0%, 62.4% (Indian).

^a^ High risk screening is not applicable to Indians as all Indians are deemed as high-risk based on UK National Institute for Health and Clinical Excellence (NICE) guidelines.
